# BEAST X for Bayesian phylogenetic, phylogeographic and phylodynamic inference

**DOI:** 10.1038/s41592-025-02751-x

**Published:** 2025-07-07

**Authors:** Guy Baele, Xiang Ji, Gabriel W. Hassler, John T. McCrone, Yucai Shao, Zhenyu Zhang, Andrew J. Holbrook, Philippe Lemey, Alexei J. Drummond, Andrew Rambaut, Marc A. Suchard

**Affiliations:** 1 Department of Microbiology, Immunology and Transplantation, Rega Institute, KU Leuven, Leuven, Belgium; 2 Department of Mathematics, School of Science and Engineering, Tulane University, New Orleans, LA USA; 3 Statistics Group, RAND Corporation, Santa Monica, CA USA; 4 Computational Biology Program, Fred Hutchinson Cancer Center, Seattle, WA USA; 5 Department of Biostatistics, Fielding School of Public Health, University of California, Los Angeles, Los Angeles, CA USA; 6 School of Biological Sciences, University of Auckland, Auckland, New Zealand; 7 Centre for Computational Evolution, University of Auckland, Auckland, New Zealand; 8 Institute of Ecology and Evolution, University of Edinburgh, Edinburgh, UK; 9 Department of Biomathematics, David Geffen School of Medicine, University of California, Los Angeles, Los Angeles, CA USA; 10 Department of Human Genetics, David Geffen School of Medicine, University of California, Los Angeles, Los Angeles, CA USA

**Keywords:** Phylogeny, Software, Statistical methods, Evolution, Phylogenomics

## Abstract

Here we present the open-source and cross-platform BEAST X software that combines molecular phylogenetic reconstruction with complex trait evolution, divergence-time dating and coalescent demographics in an efficient statistical inference engine. BEAST X significantly advances the flexibility and scalability of evolutionary models supported. Novel clock and substitution models leverage a large variety of evolutionary processes; discrete, continuous and mixed traits with missingness and measurement errors; and fast, gradient-informed integration techniques that rapidly traverse high-dimensional parameter spaces.

## Abstract (web summary)

BEAST X advances Bayesian phylogenetic, phylogeographic and phylodynamic analysis by incorporating a broad range of complex models and leveraging advanced algorithms and techniques to boost statistical inference.

## Main

The Bayesian evolutionary analysis sampling trees (BEAST) platform stands as one of the leading inference tools across a range of biological fields from systematic biology to molecular epidemiology of infectious diseases. BEAST’s success arises from its focus on sequence, phenotypic and epidemiological data integration along time-scaled phylogenetic trees. Motivation for BEAST development builds from the rapid growth of pathogen genome sequencing to deliver real-time inference for the emergence and spread of rapidly evolving pathogens to better understand their epidemiology and evolutionary dynamics. Recent scientific successes using the BEAST platform uncover the origins, spread and persistence of multiple Ebola virus outbreaks^1^, severe acute respiratory syndrome coronavirus 2 (SARS-CoV-2) variants^2^ and mpox virus lineages^3^.

BEAST X introduces salient advances over previous software versions^4^ by providing a substantially more flexible and scalable platform for evolutionary analysis with a strong focus on pathogen genomics. Two thematic thrusts describe these advances: state-of-science, high-dimensional models span multiple biological and public health domains including sequence evolution, phylodynamics and phylogeography, while new computational algorithms and emerging statistical sampling techniques notably accelerate inference across this collection of complex, highly structured models.

BEAST X incorporates new extensions to existing substitution processes to model additional features affecting sequence changes. These include a covarion-like Markov-modulated extension that incorporates site- and branch-specific heterogeneity by integrating over candidate substitution processes to capture different selective pressures over site and time^5^. Random-effects substitution models extend common continuous-time Markov chain (CTMC) models into a richer class of processes capable of capturing a wider variety of substitution dynamics, enabling a more appropriate characterization of underlying substitution processes^6^. To enable scaling of sampling-based inference under such models for large trees and state spaces, BEAST X now includes fast approximate likelihood gradients for all unknown substitution model parameters^7^. We refer to Methods for additional details regarding these substitution models.

BEAST X complements flexible sequence substitution models with advanced extensions to nonparametric tree-generative coalescent models that correct for preferential sequence sampling as a function of time^8^ and high-dimensional episodic birth–death sampling models^9^. Across tasks, BEAST X enables flexible trait evolution modeling for larger numbers of complex traits. Popular relaxed clock models capture various sources of rate heterogeneity on the phylogenetic tree, but their large numbers of model parameters can make inference difficult. BEAST X improves the classic uncorrelated relaxed clock model with a time-dependent evolutionary rate extension that accommodates rate variations through time^10^, a newly developed, continuous random-effects clock model^11^ and a more general mixed-effects relaxed clock model^12^. BEAST X enhances the previously computationally infeasible classic random local clock (RLC) model with a tractable and interpretable shrinkage-based local clock model^13^. We refer to Methods for additional details regarding these molecular clock models.

**Fig. 1:**
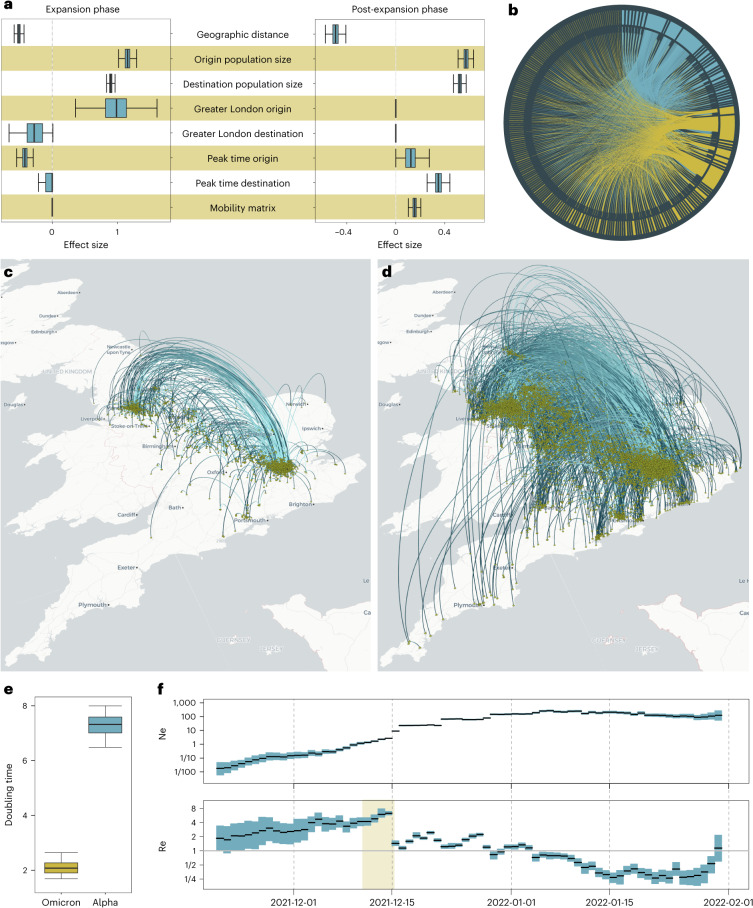
Phylodynamic analysis of the SARS-CoV-2 Omicron BA.1 invasion in England. **a**,**b**, A summary of estimates from a simultaneous estimation of sequence and discrete (geographic) trait data with a GLM extension of the discrete-trait model and two epochs (with 25 December 2021 as the transition time), showing the effect size estimates for a subset of GLM predictors considered^2^ for both epochs (**a**) and the mean Markov jump estimates between 256 lower-tier local authorities (LTLAs) for the expansion phase epoch, ordered in a clockwise fashion first by being part of Greater London (blue) or not (yellow) and then by population size (**b**). **c**,**d**, A spread.gl^30^ visualization of a RRW model fit to latitudes and longitudes (randomly drawn from within the LTLA of sampling) for the same large transmission lineage. Part of the maximum clade credibility tree is projected up to 13 December 2021 (**c**) and 25 December 2021 (**d**), respectively. The arcs represent dispersal events with a light- and dark-blue color from origin to destination, respectively. **e**, Comparison of doubling-time estimates based on an exponential growth coalescent model applied to about 1,000 genomes sampled during the expansion phase of Omicron BA.1 and Alpha (B.1.1.7) in England based on 10,000 posterior samples. **f**, Summary of estimates of effective population size (Ne) under a nonparameteric skygrid coalescent model^28^ and estimates of the effective reproduction number (Re) under an episodic birth–death sampling model^9^, based on 6,000 and 3,000 posterior samples, respectively. The yellow-shaded area in the Re plot represents the time period during which ‘Plan B’ measures were implemented in the UK. Box plots in **a** and **e** show the median (middle quartile) as a thick line, the box represents the upper and lower quartiles, and the whiskers indicate the 95% highest posterior density interval, whereas **f** shows the 95% credible intervals in blue and the median in black.

These advances underpin fast, flexible phylogeographic modeling in BEAST X. Discrete-trait phylogeography through CTMC modeling^14^ remains an attractive and widely used inference methodology. Geographic sampling bias sensitivity of the CTMC model is a common concern in phylogeographic analyses^15^. Although helpful, structured coalescent models fail to completely account for such bias^16^. BEAST X solves this problem with novel modeling^17^ and computational inference strategies: when parameterizing between-location transition rates as log-linear functions of environmental or epidemiological predictors^18^, missing predictor values often arise for one or more location pairs. BEAST X integrates out missing data within the Bayesian inference procedure by using a new Hamiltonian Monte Carlo (HMC) approach to jointly sample all missing predictor values from their full conditional distribution^2^. Figure 1 illustrates discrete-trait phylogeographic and phylodynamic analyses of SARS-CoV-2 enabled by these BEAST X advances, focusing on the Omicron BA.1 invasion in England^2^.

Continuous-trait phylogeography using relaxed random walk (RRW) models^19^ requires precise spatial location data for sampled sequences. Low-precision geographic location data represent a major barrier for spatially explicit phylogeographic inference of fast-evolving pathogens. A new approach^20^ incorporates heterogeneous prior sampling probabilities—informed by external data such as outbreak locations—over a collection of subpolygons that make up a geographic area. BEAST X now also defines homogeneous and heterogeneous prior ranges of sampling coordinates^21^. At the same time, BEAST X responds to computational challenges associated with learning branch-specific rate multipliers for large datasets by incorporating a scalable method to efficiently fit RRWs and infer their branch-specific parameters in a Bayesian framework through HMC sampling^22^.

Additional significant modeling enhancements include the scalable incorporation of general Gaussian (for example, Ornstein–Uhlenbeck) trait-evolution models^23^, missing-trait models^24^, phylogenetic factor analysis^25^ and phylogenetic multivariate probit^26^ within BEAST X. In particular, these methods successfully model dependencies between high-dimensional trait data with dozens or even thousands of observations per taxon with the help of novel computational inference techniques.

Newly introduced preorder tree traversal algorithms in BEAST X enable many of the advances we describe above. Let *N* denote the number of taxa, or leaves, on a tree. Preorder tree traversal algorithms complement their postorder counterparts and calculate vectors of partial likelihoods (for discrete traits) and sufficient statistics (for continuous traits) for each branch. With the pre- and postorder vectors together, one calculates derivatives to give rise to linear-in-*N* evaluations of high-dimensional gradients for branch-specific parameters of interest (for example, evolutionary rates of discrete/continuous traits and divergence times)^11,13,22–27^. These scalable, high-dimensional gradients enable much higher performance Markov-chain Monte-Carlo transition kernels to efficiently simulate phylogenetic, phylogeographic and phylodynamic posterior distributions.

Linear-in-*N* gradient algorithms enable high-performance HMC transition kernels to sample from high-dimensional spaces of parameters that were previously computationally burdensome to learn. BEAST X implements linear gradients with HMC for a broad collection of gold-standard models: the nonparametric coalescent-based skygrid model^28,29^ now scalably infers past population dynamics without strong assumptions regarding population size trends; mixed-effects and shrinkage-based clock models improve classic uncorrelated relaxed and random local clock models by incorporating biologically rich features to capture rate heterogeneities^11^; a variety of new continuous-trait evolution models learn branch-specific rate multipliers^22,24,26^; and novel divergence-time models efficiently overcome complex node-height restrictions by operating in a transformed space^27^. Despite the increased computational cost of gradient evaluations, Table 1 shows that applications of these linear-time HMC samplers achieve substantial increases in effective sample size (ESS) per unit time compared with the conventional Metropolis–Hastings samplers that previous versions of BEAST provide^7,9,11,24,27^. Note that these speedups are indicative and can be sensitive to the size (for example, number of taxa and number of sites; Table 1) and nature of the dataset, and to the tuning of the HMC operations. While many of the models in BEAST X already use HMC transition kernels (Table 1), ongoing developments target further extending the list of models supported by HMC. In addition, development and integration of novel and existing evolutionary, clock and coalescent models warrant continued efforts into designing and fine-turning accompanying HMC transition kernels. Specific to the data analysis presented here will be an increased focus on designing phylogeographic model formulations that are better suited to accommodating sampling bias and that offer more flexibility in their generalized linear model (GLM) extension.

**Table 1:** Performance benchmarks of HMC transition kernels

Model	Number of taxa	Number of sites	ESS speedup
Birth–death model^9^	274	918	277×
Divergence time^27^	47	2,811	7×
	211	3,186	8×
	132	11,329	14×
Substitution model^7^	583	29,903	15×
Molecular clock^11,13^	104	11,029	34×
	211	3,186	20×
	352	10,173	16×
Discrete trait^7^	1,531	1	34×
Continuous trait^24,26^	1,536	3	65×
	3.649	8	400×
Trait correlation^26^	535	24	5×

Relative speedup in terms of minimum ESS per unit time of HMC transition kernels from BEAST X over univariate transition kernels from previous versions of BEAST^7,9,11,13,24,26,27^, for datasets of different dimensions (traits instead of sites for continuous traits).

## Methods

### Substitution models

Among the newly developed and incorporated substitution models in BEAST X are Markov-modulated models (MMMs), which constitute a class of mixture models that allow the substitution process to change across each branch and for each site independently within an alignment^5^. To this end, MMMs are made up of a number of *K* substitution models (for example, nucleotide, amino acid or codon models) of dimension *S* to construct the *KS* × *KS* instantaneous rate matrix used in calculating the observed sequence data likelihood. This augmented dimensionality of MMMs leads to increased computational demands that we mitigate through recent developments in BEAGLE, a high-performance computational library for phylogenetic inference^31^. MMMs have been shown to readily integrate with Bayesian model selection through (log) marginal likelihood estimation, to substantially improve model fit compared with standard CTMC substitution models and to impact phylogenetic tree estimation in examples from bacterial, viral and plastid genome evolution^5^.

Random-effects substitution models form another extension of standard CTMC models that incorporate additional rate variation by representing the original (base) model as fixed-effect model parameters and allow the additional random effects to capture deviations from the simpler process, thereby enabling a more appropriate characterization of underlying substitution processes while retaining the basic structure of the base model that may be biologically or epidemiologically motivated^6^. Given that random-effects substitution models are in general overparameterized and, as such, not identifiable by the observed sequence data likelihood alone, one may use shrinkage priors to regularize the random effects, pulling them (often strongly) to be near or equal to 0 when the data provide little or no information to the contrary and, otherwise, attempting to impart little bias into the posterior. Further, shrinkage priors also aid in the performance of model selection, determining whether a particular random effect should be excluded from the model. One may use these models to study the strongly increased rate of C → T substitutions over the reverse T → C substitutions in SARS-CoV-2, a phenomenon that is a violation of the common phylogenetic assumption of reversibility that the majority of standard CTMC substitution models make. Applied to a dataset of 583 SARS-CoV-2 sequences, an HKY model with random effects exhibits strong signals of nonreversibility in the substitution process, and posterior predictive model checks clearly show it to be a more adequate model than a reversible model^7^.

### Molecular clock models

Recently developed molecular clock models include a time-dependent evolutionary rate model that accommodates evolutionary rate variations through time^10^. Such a phenomenon is now widely recognized in various organisms, with particular prevalence in rapidly evolving viruses that have a relatively long-term transmission history in animal and human populations^32^. This novel molecular clock model builds upon phylogenetic epoch modeling^33^ to specify a sequence of unique substitution processes throughout evolutionary history, one for each of *M* discretized time intervals in the epoch structure determined by boundaries at times *T*_0_ < *T*_1_ < … < *T*_*M*−1_ < *T*_*M*_, where *T*_*M*_ = *∞*. In this structure, the boundaries *T*_1_ to *T*_*M*−1_ determine a shift in evolutionary rate that simultaneously applies to all lineages in the tree at that point in time. This model uncovers a strong time-dependent effect that implies rate variation over four orders of magnitude in both foamy virus co-speciation and lentivirus evolutionary histories. The model improves node height (that is, time to most recent common ancestor) estimation and readily integrates with Bayesian model selection through (log) marginal likelihood estimation, where the inclusioin of time dependence yields a better fit to the data compared with other molecular clock models^10^.

A novel continuous random-effects relaxed clock model offers an alternative parameterization to the standard uncorrelated relaxed clock model^11^. In this model, the evolutionary rate *r*_*i*_ on branch *i* follows

$$\log {r}_{i}={\beta }_{0}+{\epsilon }_{i},$$

where *β*_0_ is an unknown grand mean representing the background rate in log-space and *ϵ*_*i*_ are independent and normally distributed random variables with mean 0 and estimable variance. A standard approach in BEAST X across molecular clock models is to make use of a conditional reference prior^34^ on the global tree-wise mean parameter $\exp ({\beta }_{0})$. This clock model leads to higher-dimensional parameter spaces than a simple strict clock model, but challenges in likelihood-based inference from these high-dimensional models have been addressed through applications in gradient-based optimization methods and HMC sampling (see below).

A more general mixed-effects relaxed clock model^12^ that combines both fixed and random effects in the evolutionary rate is also available, with the evolutionary rate parameter *r*_*i*_ on branch *i* now expanding to

$$\log {r}_{i}={\beta }_{0}+\mathop{\sum }\limits_{j=1}^{p}{X}_{ij}{\beta }_{j}+{\epsilon }_{i},$$

where *β*_*j*_ is the estimated effect size of the *j*th covariate *X*_*ij*_ (out of *p* covariates). For example, modeling a clade-specific rate effect with coefficient *β*_*j*_, one would set *X*_*ij*_ = 1 for all branches encompassed by the clade and *X*_*ij*_ = 0 for all other branches. This mixed-effects model has been used to confirm considerable rate variation among HIV-1 group M subtypes that cannot be adequately modeled by uncorrelated relaxed clock models, also yielding a time to the most recent common ancestor of HIV-1 group M that is earlier than the uncorrelated relaxed clock estimate for the same dataset.

Finally, the original RLC model has been reparameterized to tackle convergence and statistical mixing issues, and to achieve scalability to phylogenies with large numbers of taxa^13^. To this end, the novel shrinkage-based RLC assumes that clock rates are autocorrelated and that the incremental differences between each clock rate and its parental clock rate are equipped with a flexible, heavy-tailed, Bayesian bridge before shrink increments of change between branch-specific clocks, thereby enabling the use of a computationally efficient sampling approach to perform inference^35^. HMC sampling is used to generate proposals in increment space, using preconditioning to improve HMC performance by rescaling increment proposals, allowing larger steps to be taken in dimensions with larger variance. This novel shrinkage-based RLC has been successfully used in problems that once appeared computationally impractical, such as the study of a heritable clock structure of various surface glycoproteins of influenza A virus in the absence of prior knowledge about clock placement^13^.

### HMC sampling

HMC constitutes a gradient-based alternative to random-walk MCMC for efficient parameter inference, yielding markedly improved parameter estimation efficiency. HMC transition kernels leverage gradients to produce distant proposals with relatively high acceptance rates for the Metropolis–Hastings–Green algorithm by exploiting numerical solutions to Hamiltonian dynamics. For observed sequence data **Y** and estimable model parameters ${\mathbf{\uptheta}} = ( \theta_1, \ldots, \theta_k )$, this requires computing a number of derivatives of the observed sequence data likelihood ${\mathbb{P}}\left({\bf{Y}}| {\mathbf{\uptheta }}\right)$ on top of already calculating ${\mathbb{P}}\left({\bf{Y}}| {\mathbf{\uptheta }}\right)$, which can be computationally demanding by itself. The gradient $\nabla {\mathbb{P}}\left({\bf{Y}}| {\mathbf{\uptheta }}\right)$ is the collection of derivatives with respect to all estimable model parameters

$$\nabla {\mathbb{P}}\left({\bf{Y}}| {\mathbf{\uptheta }}\right)={\left(\frac{\partial }{\partial {\theta }_{1}}{\mathbb{P}}\left({\bf{Y}}| {\mathbf{\uptheta }}\right),\ldots ,\frac{\partial }{\partial {\theta }_{k}}{\mathbb{P}}\left({\bf{Y}}| {\mathbf{\uptheta }}\right)\right)}^{{\prime} },$$

where the prime symbol denotes the transpose operator. As with computing ${\mathbb{P}}\left({\bf{Y}}| {\mathbf{\uptheta }}\right)$, a pruning algorithm can be used to simplify calculating a single entry in $\nabla {\mathbb{P}}\left({\bf{Y}}| {\mathbf{\uptheta }}\right)$ through postorder traversal, but the ${\mathcal{O}}(NK)$ computational demands across all entries for HMC remain much higher than for standard transition kernels when *K* → *N* as for many clock models. That said, a linear-time algorithm for ${\mathcal{O}}(N)$-dimensional gradient evaluation by complementing the postorder traversal with its corresponding preorder traversal renders these computations feasible on central processing units (CPUs)^11^. Additional development of novel massively parallel algorithms enables taking advantage of graphics processing units (GPUs) to obtain further speedups over the CPU implementation^36^.

We have developed and implemented into BEAST X a wide range of HMC transition kernels that have led to drastic improvements in parameter estimation efficiency (see the main text for results)^7,9,11,13,24,26,27^. Given the increased amount of sequence data and associated metadata to be analyzed in Bayesian phylodynamic inference, HMC transition kernels are essential building blocks that enable complex Bayesian phylodynamic analyses in a reasonable amount of time. This is illustrated in the following section’s practical example, where the combination of a large number (11,351) of taxa from 256 discrete geographic locations (the upper limit in the current computational architecture and implementation in BEAGLE^31^) would be completely infeasible to analyze without the help of HMC.

### Application to SARS-CoV-2

Figure 1 illustrates a variety of advances in BEAST X modeling and inference strategies as applied to the phylogeographic and phylodynamic analysis of SARS-CoV-2. Specifically, it focuses on phylodynamic analyses of the Omicron BA.1 invasion in England^2^. Figure 1a reports effect size estimates for covariates in a GLM extension of discrete phylogeographic inference for the largest BA.1 transmission lineage identified by Tsui et al.^2^ (11,351 genomes). The phylogeographic inference involved two epochs^33^ to estimate separate covariate effect sizes in the expansion and postexpansion phase, showing, for example, differences in dispersal out of Greater London and in contributions of mobility. The epoch-GLM discrete diffusion model was fit to a set of trees estimated using the Thorney BEAST approach^37^, inferring dispersal among 256 lower-tier local authorities (LTLAs) in England. HMC inference was required to fit this high-dimensional diffusion model while integrating over some degree of missing data in the covariates, and the computation benefits tremendously from using GPUs^31,36^. In addition to effect size estimates illustrated here, Tsui et al.^2^ introduce a new estimate of relative predictor importance-based deviance measures. Figure 1b summarizes Markov jump estimates^38^ between the LTLAs during the expansion phase, showing dispersal from Greater London LTLAs (in blue) as well as from other LTLAs (in yellow). Figure 1c,d illustrates spatiotemporal projections of a maximum clade credibility summary of a continuous phylogeographic inference of the same large BA.1 transmission lineage, including a mapping of the first half (Fig. 1c) and the complete expansion phase (Fig. 1d). This visualization was achieved using spread.gl^30^, a high-performance browser application that uses the kepler.gl framework to accommodate large-scale data. Fitting the RRW model in this analysis required HMC inference to efficiently integrate over the branch-specific rates of diffusion^22^. Using standard Metropolis–Hastings kernels on the RRW branch rates does not complete MCMC burn-in in the same compute-time it takes HMC to deliver ESSs >100 across all 22,700 rate parameters from the converged posterior distribution. Figure 1e,f represents estimates of transmission dynamics using several tree generative priors. We compare doubling-time estimates for a subset of genomes representative for the expansion phase of the largest BA.1 transmission lineage (*n* = 1,000) to a genomic dataset representative for expansion of the Alpha variant in England (*n* = 976)^39^. This highlights an Omicron BA.1 doubling time that is about 3.5 times smaller compared with Alpha. We include estimates of effective population size (Ne) through time using a nonparametric coalescent model inferred from the set of empirical trees with 11,351 tips, showing a roughly linear increase in log Ne in the expansion phase as opposed to roughly contant log Ne in the postexpansion phase. Finally, we provide comparative estimates of the effective reproduction number (Re) through time using an episodic birth–death sampling model fit to the same set of empirical trees^9^, showing a considerable decrease in Re after implementation of measures against the spread of Omicron in the UK.

### Reporting summary

Further information on research design is available in the Nature Portfolio Reporting Summary linked to this article.

## Online content

Any methods, additional references, Nature Portfolio reporting summaries, source data, extended data, supplementary information, acknowledgements, peer review information; details of author contributions and competing interests; and statements of data and code availability are available at 10.1038/s41592-025-02751-x.

## Supplementary information

Reporting Summary

## Data Availability

All data required to perform the analyses in Fig. 1 are available as XML input files for BEAST X via GitHub at https://github.com/beast-dev/beast-mcmc/tree/master/examples/BEASTXRelease.

## Code availability

By naming this major release BEAST X v10.5.0, we delineate our platform from the related but independent BEAST 2 project^40^. The jump from BEAST v1.10.5 to BEAST X v10.5.0 facilitates best-practice semantic versioning and clarifies continued active development. BEAST X is open-source under the GNU lesser general public license and available via GitHub at https://beast-dev.github.io/beast-mcmc for cross-platform compiled programs and https://github.com/beast-dev/beast-mcmc for software development and source code. It requires Java version 1.8 or greater and the platform also depends on the BEAGLE high-performance phylogenetic likelihood library version ≥4.0. BEAST X interfaces with recent releases of the BEAGLE high-performance phylogenetic likelihood library^31^ to off-load computationally expensive calculations to multicore CPUs and GPUs on laptop, desktop and cluster devices. These calculations include for the first time the preorder traversals^36^ necessary for linear-time evaluation of high-dimensional gradients of the sequence and trait data likelihoods. Documentation, tutorials and help are available at http://beast.community, and many users actively discuss BEAST usage and development in the ‘beast-users’ GoogleGroup discussion group (http://groups.google.com/group/beast-users).
